# Lentiviral Vector Pseudotypes: Precious Tools to Improve Gene Modification of Hematopoietic Cells for Research and Gene Therapy

**DOI:** 10.3390/v12091016

**Published:** 2020-09-11

**Authors:** Alejandra Gutierrez-Guerrero, François-Loïc Cosset, Els Verhoeyen

**Affiliations:** 1 Gastroenterology and Hepatology Division, Joan and Sanford I. Weill Department of Medicine, Weill Cornell Medicine, Cornell University, New York, NY 10021, USA; gutierrezgalejandra@gmail.com; 2 The Jill Roberts Institute for Research in Inflammatory Bowel Disease, Weill Cornell Medicine, Cornell University, New York, NY 10021, USA; 3 CIRI, Université de Lyon, INSERM U1111, ENS de Lyon, Université Lyon 1, CNRS, UMR 5308, 69007 Lyon, France; flcosset@ens-lyon.fr; 4 INSERM, C3M, Université Côte d’Azur, 06204 Nice, France

**Keywords:** lentiviral vector, gene therapy, T cell, B cell, HSC, CRISPR/CAS9, NK cell, pseudotyping

## Abstract

Viruses have been repurposed into tools for gene delivery by transforming them into viral vectors. The most frequently used vectors are lentiviral vectors (LVs), derived from the human immune deficiency virus allowing efficient gene transfer in mammalian cells. They represent one of the safest and most efficient treatments for monogenic diseases affecting the hematopoietic system. LVs are modified with different viral envelopes (pseudotyping) to alter and improve their tropism for different primary cell types. The vesicular stomatitis virus glycoprotein (VSV-G) is commonly used for pseudotyping as it enhances gene transfer into multiple hematopoietic cell types. However, VSV-G pseudotyped LVs are not able to confer efficient transduction in quiescent blood cells, such as hematopoietic stem cells (HSC), B and T cells. To solve this problem, VSV-G can be exchanged for other heterologous viral envelopes glycoproteins, such as those from the Measles virus, Baboon endogenous retrovirus, Cocal virus, Nipah virus or Sendai virus. Here, we provide an overview of how these LV pseudotypes improved transduction efficiency of HSC, B, T and natural killer (NK) cells, underlined by multiple in vitro and in vivo studies demonstrating how pseudotyped LVs deliver therapeutic genes or gene editing tools to treat different genetic diseases and efficiently generate CAR T cells for cancer treatment.

## 1. Introduction

Viral vectors have been derived from a large number of viruses, which were transformed into efficient tools for delivery of genes into cells of interest, thanks to their unique characteristics. Viruses have undergone modifications in their replication pathway to reduce risks when used for therapeutic purposes. Each vector system offers advantages and limitations as no system offers a 100% efficiency for all cell types. They can be classified by their capacity or not to integrate into the host cell genome. The adeno-associated viruses (AAV) and adenoviruses (AdV) [1] are non-integrative, while the *Retroviridae* family, such as foamy virus [2], murine leukemia virus (MLV) or human immunodeficiency virus (HIV), among others [1] are integrative.

Retrovirus-based vectors, MLV-derived vectors in particular, were among the first to be developed in the 80s and 90s [3]. However, in recent years the number of clinical trials in which they are employed has been reduced to a 0.5% in contrast to 11 years ago when MLV-derived vectors accounted for 21% of the clinical trials in gene therapy. On the other hand, the number of clinical trials which include lentiviral vectors (LVs) has increased from 1.4% to 10% [4].

Viral vectors have been used in clinical trials for more than 20 years, they include all types of integrative and non-integrative vectors (e.g., MLV, LV, AAV, AdV) [5]. To choose the appropriate vector, we must take into consideration numerous factors; target tissue or cell, viral genome packaging capacity, propensity to immunotoxicity, tropism, in vivo or ex vivo delivery and potential of genomic integration or not.

In this review, we will focus on LVs, their optimization by pseudotyping with heterologous viral envelopes and their applications for gene therapy using different primary cell types.

### Lentiviral Vectors

**Figure 1:**
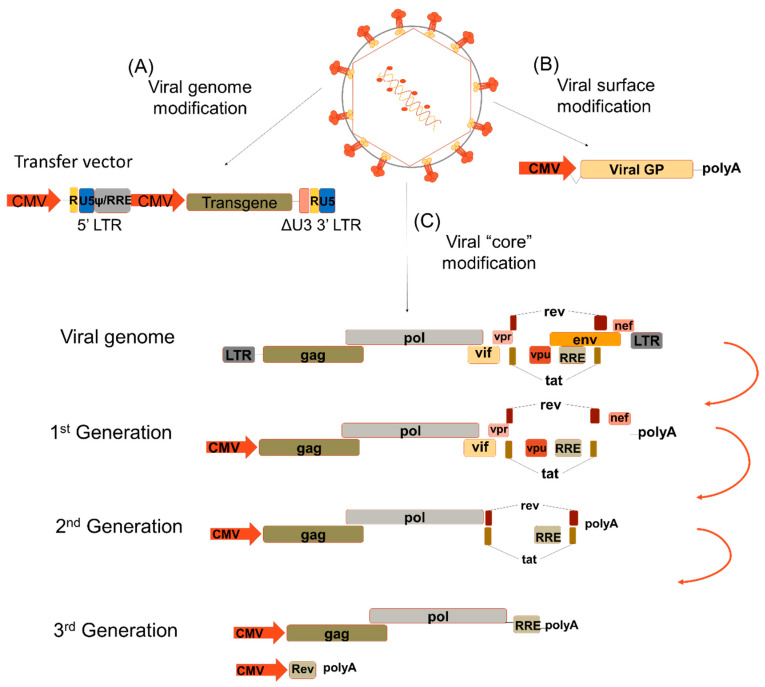
Lentiviral modifications. (**A**) The transfer vector contains the long terminal repeats (LTR) and the transgene is under a strong internal promoter, i.e., CMV. (**B**) The viral surface proteins are exchanged by different viral glycoproteins to confer them a different tropism according to the cell targeted for transduction. (**C**) The viral genome encodes three genes flanked by LTRs: structural (gag, pol and env), regulatory (rev and tat) and accessory (vif, vpr, vpu and nef). The 1st generation lentiviral vectors (LVs) contained all the viral proteins with the exception of the Env protein. The 2nd generation LV does not express any of the accessory proteins. The 3rd generation LV is divided in two; one expresses the structural proteins gag and pol while the other expresses the regulatory protein rev. LTR—long-terminal repeats; U5—5′UTR; U3- 3′UTR; ψ—Psi packaging element; RRE—Rev response element; CMV—cytomegalovirus; Viral GP—viral glycoprotein; gag—group-specific antigen; pol—DNA polymerase; env—viral envelope; rev- transactivating protein; tat—trans-activator of transcription; vif—viral infectivity factor, vpr—viral protein R; vpu—viral protein u; nef—negative regulatory factor.

LVs have been selected as a tool for gene delivery due to their ability to transduce all type of non-diving [6] or slowly proliferating cells making them very attractive for clinical applications. LVs are part of the *Retroviridae* family together with the gamma-retroviruses. They contain an RNA genome that is retrotranscribed to DNA in the transduced cell [7]. The first generation of retroviral vectors set the basis of important principals to ensure safe use of these vectors. Firstly, there is a potential of recombination events during manufacturing of the vectors that could results in replication-competent virus [7]. To avoid this, there was a need for splitting the viral genome into different expression constructs. Secondly, the enhancer and promoter sequences from the long terminal repeats (LTRs) were deleted to generate what is called self-inactivated (SIN) vectors; this is a safety measure to avoid activation of surrounding (onco-)genes as already reported in some clinical trials with γ-retrovirus vectors [8,9]. Thirdly, the incorporation of heterologous envelope glycoprotein proteins onto the vector surface will expand or restrict the host range of the vector, a process called pseudotyping [6] (Figure 1).

In clinical trials, AAVs are chosen for in vivo gene transfer, while LVs are up to now the preferred tools for ex vivo gene correction [10]. Their main advantage is that they are derived from viruses that have been selected by evolution for transducing human cells, however, this also has led to protection against these viruses and the vectors derived from these viruses by the human immune system. Some components of viral vectors are highly conserved, which helps the human immune system to recognize and destroy them. Therefore, immune-mediated rejection is one of most significant obstacles in gene transfer in human cells, particularly in vivo. Of note, the human immune system acts very differently to different vector types [10].

Other obstacles have been encountered, such as horizontal and/or vertical transmission, when the transferred gene could pass to someone we share the household with or to an offspring. Genotoxicity, when there is an overexpression or dysregulation of the expression of the transgene. Gene silencing has been observed in in vitro studies [11], but has only been observed in rare cases in vivo. For example, in one report, low efficiency of transgene expression was observed in the first clinical trials for chronic granulomatous disease (CGD) (reviewed in [12]), which was caused by silencing. Immunotoxicity and genotoxicity have been observed in different clinical trials (review in [5]). Immunotoxicity—when the viral vector induces a response from the immune system—has been reported for AAV upon its in vivo delivery in different diseases such as hemophilia, spinal muscular atrophy, neuropathies and dystrophies [5]. Genotoxicity, one of the most problematic events caused by integration of the vector sequences into the host cell genome, can result in malignant cell transformation. This is called insertional mutagenesis—disruption or upregulation of genes due to the viral integration event—and causes outgrowth of clones in a polyclonal population [6]. This was the case for different gene therapy clinical trials using gamma retroviral vectors such as X-linked severe combined immunodeficiency (SCID-X1) [13,14], CGD [15] and Wiskott–Aldrich syndrome (WAS) [16]. In more recent clinical trials that used LVs, a more polyclonal pattern of corrected hematopoietic cells has been observed [17,18,19,20].

LVs underwent “generations” of modifications. They are classified according to the packing plasmid used for their production. The first generation included the HIV gag, pol, regulatory genes and accessory genes. The second generation was able to dispose of the accessory genes without any negative effect on their infectivity or vector yield. Additionally, they improved in safety since any replication-competent lentivirus formed was devoid of virulent factors. With the third generation, the safety was further improved as the promoter in the LTR was now rendered independent of the HIV protein TAT for its activation (Figure 1). An extra safety measure consisted in inactivating the integrase without affecting reverse transcription and transport to the nucleus. These non-integrative LVs have been used efficiently in post-mitotic tissues, especially in combination with gene editing tools (zinc finger nucleases (ZFN), transcription activator-like effector nucleases (TALENs) and clustered regularly interspaced short palindromic repeats (CRISPR)/CRISPR-associated 9 (Cas9); see point 4) [4,21,22].

For the LV to deliver its cargo into a cell, it needs to interact with a cellular receptor to trigger the fusion of the viral envelope with the cell membrane. This tropism is defined by the viral envelope glycoprotein. However, the wild type HIV glycoprotein, gp120, has very specific tropism for human CD4+ T cells and monocytes and produced low titer vectors. The solution is to exchange the HIV envelope glycoprotein for heterologous glycoproteins on the LV surface, a process called pseudotyping [4].

## 2. Pseudotyping

Pseudotyped LVs consist of virus particles bearing glycoproteins derived from other enveloped viruses, conferring the LVs, the tropism of the virus from which the glycoprotein was borrowed. It was Page et al. in the 90s, who first designed and tested a HIV-based vector containing a heterologous glycoprotein. In a first attempt, they replaced the original HIV envelope glycoprotein with the MLV envelope glycoprotein and produced infectious vectors [23]. It was only 6 years later that 3 groups [24,25,26] showed that the vesicular stomatitis virus G glycoprotein (VSV-G) was efficiently incorporated into HIV vectors. This was a considerable step forward since VSV-G appeared to interact with an ubiquitous cellular receptor, a phospholipid component of the cell membrane [27,28], which conferred the ability of entry into multiple cell types tested and conferred high stability to the infectious vector particles [29]. This broad tropism included non-proliferating cells such as hematopoietic stem cells (HSC), the target cell par excellence in gene therapy. VSV-G pseudotyped LVs possess other important characteristics as a delivery vector, such as resistance to freeze-thaw cycles and ultracentrifugation [30,31], which allow to improve vector titers. On the other hand, there are some drawbacks in using VSV-G pseudotyped LVs. The wide cell tropism can lead to binding to the surface of any cell encountered before reaching its target cells. Moreover, it has been shown that the VSV-G pseudotyped LVs are inactivated by human sera from some donors, preventing its effective use for in vivo gene delivery [30,32,33]. VSV-G is also cytotoxic when expressed stably in human cells, which did not permit the development of stable LV producer cell lines [32,33]. Additionally, there is a strong possibility that humans develop a potent immune response against VSV-G after administration [33], which would restrict the efficacy as well as future inoculations of other VSV-G pseudotyped LVs into a patient.

The Indiana strain of the vesicular stomatitis virus is still the standard envelope used for pseudotyping. It recognizes the low-density lipoprotein receptor (LDL-R) for attachment and entry into the cell. However, it has been shown that VSV-G needs to traffic through the endosomal network of the cell and requires a low pH to fuse and eject its LV content into the cytoplasm before the viral RNA can be retrotranscribed and migrate into the nucleus and integrate [34,35]. It is the variation in the levels of the LDL-R expression that explains the low efficiency of the LVs pseudotyped with VSV-G in certain cell types. For example, the levels of LDL-R in unstimulated human T, B and hematopoietic stem and progenitor cells (CD34^+^ cells) are very low. Stimulation of the T cell through the T-cell receptor or the CD34^+^ cells with cytokines upregulated LDL-R expression and permitted transduction of these cells with VSV-G pseudotyped LVs. In contrast, activation of B cells only mildly upregulated LDL-R, leaving this target cell poorly permissive to VSV-G pseudotyped LVs [36].

There are many other viral glycoproteins that have been incorporated into LVs to improve their infectivity and confer them a more selective tropism, like the gibbon ape leukemia virus (GALV) or the cat endogenous retrovirus envelopes (RD114) [30]. H and F envelope proteins from the measles virus (MV) have been used to retarget various cell types. More recently other proteins have been considered as alternative envelopes for LV pseudotyping such as glycoproteins derived from other vesiculovirus subfamilies, Cocal, Piry and Chandipura viruses or the VSV New Jersey strain as well as the Nipah virus and other rhabdoviruses, for which it was proposed that they could have an advantage over the commonly used VSV-G Indiana strain [37,38]. However, it is difficult to obtain high-titer vectors with some of these glycoproteins, though they transduce efficiently hematopoietic cells (Summary in Table 1).

### 2.1. Pseudotyping of LVs with Baboon Endogenous Virus and Feline Endogenous Virus

The baboon endogenous virus (BaEV) is an endogenous gammaretrovirus initially isolated in the 70s from a baboon placenta and was then cocultivated with a human rhabdomyosarcoma cell line (A204). It is a recombinant virus between a *Papio cynocephalus* endogenous retrovirus and a simian betaretrovirus [51]. It has been shown that BaEV is intimately related with an infectious feline endogenous retrovirus (RD114). The *env* gene from the RD114 is thought to be originally derived from the BaEV envelope gp. These two viruses are stable in human and macaque sera, giving them a great potential for in vivo gene therapy. They also recognize the sodium-dependent neutral amino acid transport (ASCT-2) in human cells, but only BaEV also recognizes ASCT-1, giving BaEV a wider tropism [47,52]. ASCT-1 and -2 receptors have a 57% identical sequence, and they are expressed in a wide number of cells.

These glycoproteins are of great interest because it has been reported that their receptors are expressed on human CD34^+^ cells, T and B cells [30,36,43,45,53,54,55]. That is why BaEV and RD114 envelopes have been used to improve LVs for the transduction of these primary cells.

The first report of an LV efficiently pseudotyped with the BaEV glycoprotein was in 2014 by Girard-Gagneapin et al. [36]. They showed a high transduction rate (60–90%) in mildly stimulated hCD34^+^ cells, and up to a 30% in unstimulated CD34^+^ cells. In the case of RD114, it was reported that pre-stimulation with cytokines increased the transduction rate due to the increase of the ASCT-2. It was later reported that BaEV pseudotyped LVs were able to transduce stimulated B cells with a very high transduction rate (up to 80%) in comparison with the VSVG-LVs resulting in low transduction levels (5%). Additionally, BaEV-LVs were able to transduce resting naïve B cells and memory B cells (20–40% efficacy) which had not been reported before [42]. In the case of T cells, recent data demonstrated that BaEV pseudotyped LVs are capable of transducing not only naïve T cells but also early thymocytes and natural killer cells with high transduction rates (up to 80%; Table 1) [39,40,41].

**Figure 2:**
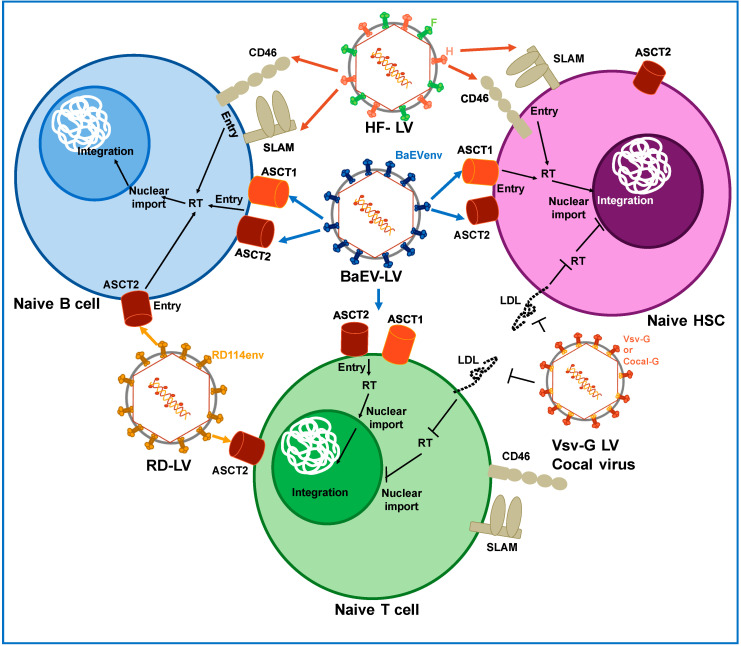
Pseudotyping of lentiviral vectors. Lentiviral vectors were generated expressing different envelopes from other viruses such as measles virus (HF), baboon envelope (BaEV) and feline endogenous retrovirus RD114 envelope gps (RD). They recognize receptors like the complement receptor CD46, signaling lymphocytic activation molecule (SLAM), sodium-dependent neutral amino acid transporters, ASCT-1 and ASCT2, expressed on resting hematopoietic stem cells (HSC), T cells and B cells. However, envelopes from vesicular virus (VSV-G) and Cocal virus recognize the low-density lipoprotein (LDL) receptor, which is poorly expressed on resting cells, which is the reason why they allow poor entry in these cells, and consequently, low level integration into the cell genome. Colored arrows indicate binding of the envelope gps to their respective receptors.

All these data suggest that BaEV pseudotyped LVs are efficient tools for modification of primary target cells in gene therapy and immunotherapy (Figure 2).

### 2.2. Pseudotyping LVs with H and F Glycoprotein from Measles Virus

MV is part of the *Paramyxoviridae* family, which contains a negative-strand RNA genome and encodes six structural proteins. The glycoprotein H and F are embedded as spikes in the membrane. The hemagglutinin (H) protein attaches to the cellular receptors and the F protein mediates fusion of the MV membrane with the host cell membrane for the delivery of all of the viral components into the host cytoplasm [56]. The MV wild type strain uses the signaling lymphocyte activation molecule (SLAM) as a receptor, whereas the laboratory and vaccine strain like the Edmonston strain gained an additional entry through the CD46 receptor, due to adaptation in SLAM-negative cells [56,57]. SLAM is expressed at the surface of T and B cells whereas CD46 is expressed by all human nucleated cells, increasing their tropism. It has been reported that H/F-LVs incorporating the H from the Edmonston vaccinal strain were able to efficiently transduce quiescent T and B cells (Figure 2). Frecha et al. [57] reported that simultaneous transduction with BaEV pseudotyped LVs and VSV-G pseudotyped LVs, did not facilitate the entry of VSVG pseudotyped LVs into T cells, emphasizing that each pseudotype used a different cell entry mechanism. They further proved that in order to obtain high transduction rates in resting T and B cells, both receptors, CD46 and SLAM, have to be correctly engaged [57].

H/F pseudotyped LVs were identified as the first tool allowing efficient transduction of resting memory and naïve T cells (up to 50% transduction) without activating or changing their cytokine profile [46]. Quiescent B cells have also been successfully transduced with H/F pseudotyped LVs at similar transduction levels as T cells [42,58]. In addition, it has been recently reported that H/F pseudotyped LVs also have the potential to transduced with a 100% efficiency pre-stimulated HSCs with low doses of H/F pseudotyped LVs. In the case of unstimulated HSCs, the transduction levels reached 70%. It was further demonstrated that these high levels were maintained or increased even after successive rounds of engraftment in NOD/SCIDγC^−/−^ (NSG) mice [47]. These results indicated that high levels of HSCs were transduced.

One variant of the BaEV glycoprotein, in which the cytoplasmic tail was switched for the one of the MLV envelope glycoproteins demonstrated no cytotoxicity upon transfection, and therefore can be considered as a viable candidate to generate stable LV packaging cell lines [36,59]. In the case of the H/F packing cells, some modifications were introduced to decrease cell toxicity, such as knocking-out the expression of CD46, the measles virus receptor, in the 293T producer cells to prevent cell to cell fusion during the vector production [31]. Nevertheless, one of the drawbacks of BaEV-LVs and H/F-LVs is that their infectious titers are lower than their VSV-G LV counterparts, which is a hurdle that needs to be overcome for future clinical applications.

### 2.3. Pseudotyping LVs with Nipah Virus Envelopes

Nipah virus (NV) is a negative-sense single-stranded RNA virus from the genus *Henipavirus* from the *Paramyxoviridae* family. It has a very broad tropism and uses protein-based receptors [50,60]. Similar to the MV envelopes, the NV envelopes encode two glycoproteins to allow entry into the cell: the attachment protein (G), which allows the virion attachment to the cellular receptors and the fusion protein (F), which mediate the union of the viral membrane with the cell membrane [49,61,62]. NV’s main receptors are ephrinB2 and ephrinB3, an alternative receptor conferring less efficient entry. EphrinB2 is strongly expressed in arterial endothelial cells, vascular smooth muscle cells, pericytes and tumor endothelium [49,50]. Moreover, ephrinB2 has been suggested as a marker for stemness, as it is expressed in murine embryonic stem cells, HSC and neural stem cells [63]. However, when NV pseudotyped LVs were tested in these cells, the percentage of transduction was very low (3.5%). They proposed that this is due to the exclusive expression of ephrinB2 in long term HSC (CD34^+^ CD38^−^ CD90^+^ cells) and percentage of this population is less than 8% of the total HSCs [50]. Nevertheless, it was shown that NV pseudotyped LVs were able to transduce primary endothelial cells very efficiency and it is proposed to be a perfect tool for in vivo gene therapy of the vascular system.

Due to the low prevalence of the NV, it was suggested that it would be highly unlikely that there would be a humoral immunity in humans when using NV envelope for LV pseudotyping [49,61].

Recently, several vaccines based on the G glycoprotein of the NV to protect against lethal infections, are evaluated in pre-clinical trials [60], underlining its capacity to induce an immune response. Even though the role of antibodies in immunization against NV infection has been extensively reported, few studies addressed the induction of T-cell immunity. Kalodimou et al. [60] have recently reported potential epitopes that stimulate antigen-specific CD4^+^ and CD8^+^ T cells. This suggests that NV pseudotyped LVs might possibly be more immunogenic than first thought.

### 2.4. Cocal Virus

Cocal virus (CV) is a member of the *Rhabdoviridae* family, genus Vesiculovirus, which in turn has been classified in two serotypes: New Jersey and Indiana. Indiana has been divided in three serological groups. The CV belongs to serological group type 2 and was isolated from rat’s mites in the 1960s in different regions in South America [64]. This glycoprotein shares 72% identity at the amino acid level with the VSV-G Indiana strain. However, it is distinct from it in the sense that it is more resistant to complement-mediated inactivation by mouse and human sera, and it also confers an even wider tropism. Furthermore, CV glycoprotein pseudotyped LVs can be produced at higher titers and can transduce not only human cells, but also nonhuman primate and canine stem cells [32,33,65].

Humbert et al. [32] were able to transduce HSC and CD4^+^ T-lymphocytes more efficiently with CV pseudotyped LVs than VSV-G pseudotyped LVs. It is not clear if other receptors are involved, if CV has more affinity with the LDL-R, is more efficient in merging with the cell membrane or if CV pseudotyped LVs display more envelope molecules per vector particle. Nevertheless, CV can be highly expressed in producer cells, and therefore represent a perfect pseudotype for engineering of a stable packaging cell line with clinically usable titers [32]. It has also been reported that CV pseudotyped LVs more efficiently transduce human CD34^+^ NOD/SCID mouse repopulating cells as well as CD34^+^ bone marrow cells with lower MOI than the counterpart VSV-G pseudotyped LVs [33].

### 2.5. Envelope Glycoproteins Retargeted to Specific Hematopoietic Cells

In order to improve the vector tropism to hematopoietic cells, another strategy is to incorporate cell targeting proteins (CTPs) into the viral envelope’s outer domain [66]. There are two main steps for virus entry: (1) virus-cell attachment and (2) fusion of virus and cellular membrane. For a number of enveloped viruses, such as MLV, BaEV and RD114 retroviruses, both receptor binding and fusion functions are not independent since they are present in a single glycoprotein. Therefore, attempts to retarget these vectors to other cell surface epitopes were not successful: though correct binding to a receptor of choice was achieved, the cell-virus membrane fusion function was abolished. Other viruses such as Nipah and MV have these two functions, receptor binding and fusion, separated in two different glycoproteins. Therefore, insertion of a CTP into the receptor binding glycoprotein allows retargeted binding to a receptor of choice without affecting fusion function. Importantly, to achieve specific retargeting of the glycoproteins to another receptor on the cell, the natural binding sites of the glycoproteins first need to be abolished.

Some of the CTP molecules used for this purpose are single-chain variable fragments (scFvs). However, this can be challenging as scFvs do not allow targeting of more than one epitope and using several scFvs will impair the folding and therefore the fusion with the cell membrane. The use of ankyrin repeat proteins (DARPins) constitutes an alternative due to their versatility and affinity. The ankyrin domains have been selected from libraries to guaranty their high affinity. ScFvs and DARPins have been introduced successfully in MV [67] and in Nipah virus envelope glycoproteins [61,68,69] to target oncolytic domains and hematopoietic cells in vitro and in vivo [48,68,70,71,72].

Morizono et al. [69] designed a pseudotyped LV able to transduce melanoma tumor cells using a similar strategy. They co-pseudotyped LVs with the Sindbis virus glycoproteins E1 fusion protein and a mutated E2 protein non-covalently linked to a specific monoclonal antibody directed against melanoma antigen. Alternatively, using Sindbis glycoproteins, Kasaraneni et al. [66] used a simple ‘plug and play’ strategy to retarget lentiviral vectors to any desired cell type through in vitro covalent modification of the vectors with specific CTPs.

## 3. Gene Therapy

### 3.1. Introduction to Gene Therapy

Gene therapy is defined as the introduction of therapeutic genes into target cells in order to treat a medical disorder or disease. It has been very relevant in monogenic disease treatments, as simply the introduction of a therapeutic gene can correct the genetic defect. The therapeutic transgene can either replace the function of the affected gene, increase the physiological production of the substance or produce the substance when missing in the target organism [73,74]. This possibility of a durable cure for life by one single application or modification has made gene therapy very attractive.

**Figure 3:**
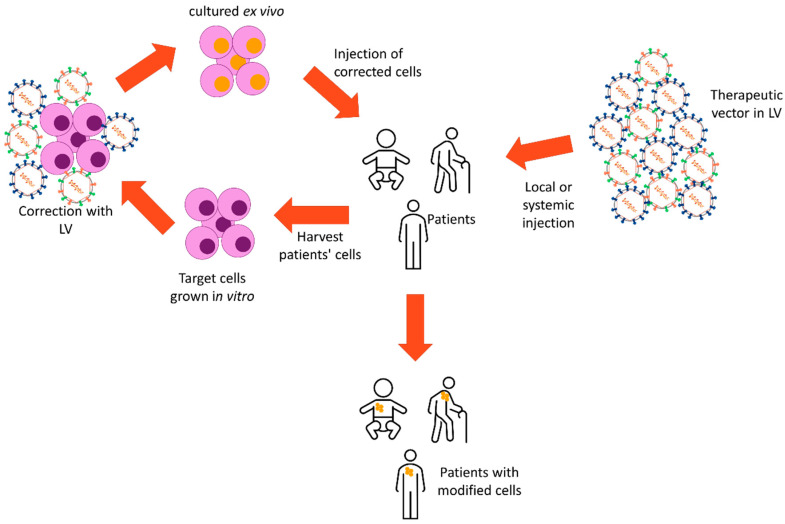
Gene therapy in vitro and in vivo. Cells from patients are harvested and cultured and modified with lentiviral vectors (LV) in vitro carrying a therapeutic vector. The corrected cells are then injected back into the patient for engraftment in order to alleviate the disease. The therapeutic vector can also be injected locally or systemically into the patients which will then transduce the target cells in vivo and correct the disease.

Gene therapy’s main challenge is to achieve a durable expression of the therapeutic gene in a large percentage of the target cells without changing their normal physiology [5,74]. The concept first appeared during the 1960s–70s where recombinant DNA techniques demonstrated that foreign genes could correct genetic defects and improve disease phenotypes in vitro [4]. The introduction of the transgene can be performed in different ways, either by taking the cells from the patient and modifying them ex vivo to then re-inject them or by directly introducing the delivery method into the patients, in vivo (Illustrated in Figure 3). It was not until 1995 when Donald B. Kohn et al. [75] showed the first result of a clinical trial where HSCs were gene corrected for Adenosine deaminase deficiency (ADA). However, the expression of the transgene was transient, and patients were unable to abandon protein replacement therapy. Nevertheless, this was a first positive step towards gene therapy which proved safe and conferred therapeutic benefit to the patient, though transient. This encouraged the field to look for improvements in vector design. In the last couple of decades, these improvements have given way to various numbers of successful clinical trials in diverse monogenic diseases, such as severe combined immunodeficiencies (SCID) and β-hemoglobinopathies [76,77], which today resulted in two dozen gene therapies, clinically approved as drugs [78].

There were successful trials reported in gene therapy for some immunodeficiencies such as SCID caused by ADA, ADA-SCID and SCID-X1 with 100% survival rates and over 80% efficiency [76,79,80]. Others, like WAS [9,16,19,81] and X-linked CGD X-CGD [82] were not as successful, showing major complications and revealed the requirement to improve vector systems [76].

Multiple adverse events have been reported in clinical trials, in most cases due to gammaretroviral vectors that caused insertional mutagenesis. Therefore, the field has focused on the development of safer vectors. In the clinical trials using lentiviral vectors, no adverse events have been reported due to insertional mutagenesis up to now. However, there is a need to improve the transduction process itself in order to increase the percentage of gene modified cells and thus efficacy of the gene therapy. Pseudotyping is playing a major role in improvement of transduction levels of hematopoietic target cells.

### 3.2. Hematopoietic Stem Cell-Based Gene Therapy

HSCs are desired target cells in gene therapy because any genetic modification will be transferred to all lineages derived from them [83]. The pseudotyped lentiviral vectors mentioned previously could be beneficial in gene therapy of different genetic diseases, with some examples such as bone marrow failures, e.g., Fanconi anemia (FA) and β-hemoglobinopathies, e.g., β-thalassemia and sickle cell disease.

FA is a rare genetic disorder characterized by progressive failure of the bone marrow. The goal of FA gene therapy is to develop an alternative treatment for this bone marrow failure and prohibit the development of leukemia or other cancers in these patients that carry a mutation in DNA repair enzymes encoded by FANC genes. Correction of HSCs or hematopoietic progenitor cells (HPC) offers a potential cure, because even with a relative low frequency of HSCs/HPCs expressing the wild type gene, one corrected HSCs is capable of repopulating the bone marrow and leading to normal hematopoiesis [84,85,86]. The main problem until recently was the efficiency of transduction of HSCs. It was shown that VSV-G pseudotyped LVs were not able to transduce HSCs efficiently because they lack LDL receptor expression in their unstimulated state [55], so there is a need for alternative vector tools.

Frecha et al. [53] generated LVs pseudotyped with RD114 envelope glycoprotein, which co-displayed HSC stimulating cytokines and showed that these LVs were able to efficiently transduce human CD34^+^ cells in both total cord blood and bone marrow. These modified CD34^+^ cells were able to colonize immunodeficient mice and moreover resulted in a selective long-term transduction of human HSCs in vivo. This is a step forward in improving the transduction of FA HSC in bone marrow ex vivo or even in vivo and improved the efficacy of FA gene therapy. More recently, H/F pseudotyped LVs successfully achieved high-level transduction of unstimulated HSC, which was maintained in all hematopoietic lineages in secondary recipient NSG mice [47]. These H/F pseudotyped LVs could be therefore beneficial for transducing CD34^+^ cells of FA patients since these rare target cells do not easily survive ex vivo culture in the presence of cytokine stimulation. For the last 15 years, FA gene therapy has been studied in multiple pre-clinical studies, but to date only one clinical trial using VSV-G pseudotyped LVs has been performed [85].

The β-thalassemia syndromes are autosomal blood disorders, characterized by the reduction or absence of the β unit of the hemoglobin [87]. The first clinical trials used a VSV-G pseudotyped LV expressing the entire β-globin showing an increase in the hemoglobin in the thalassemia patients. However, there is a need to improve the efficacy of gene correction in the HSCs from these patients. In recent in vitro studies, it has been shown that incorporating some enhancer elements into the vector combined with use of the BaEV envelope glycoprotein for LV pseudotyping increased transduction efficiency at low MOIs in comparison with the VSV-G pseudotyped LV and resulted in a more stable and high expression of the hemoglobin [88].

The examples presented above emphasize again the importance of using an adequate envelope for LV pseudotyping in order to achieve high transduction rates of patient HSCs.

### 3.3. Gene Therapy Using T Cells

#### 3.3.1. Why Are T Cells Important Target Cells for Gene Therapy?

Efficient gene transfer into T lymphocytes may allow treatment of a number of genetic dysfunctions of the hematopoietic system, such as immunodeficiencies as well as de development of novel therapeutic strategies for cancer and acquired immunodeficiency syndromes [89,90]. The main benefit of T cells is that they are more accessible to genetic modifications, can normally be isolated in high amounts and have lower risk of transformation, as no leukemia has been observed in T cell-based gene therapy [89,91]. Naïve T cells are a desirable target as they can respond to novel antigens and have a long-term lifespan which allows them to survive for years in patients. Moreover, CD4^+^ T cells are considered key players in coordinating the immune response as they interact with CD8^+^ T cells, B cells and dendritic cells, so they become an important target not only for gene therapy and immunotherapy approaches but also in fundamental immunology [48]. In gene therapy, the main goal is to preserve the T-cell phenotype and their properties to react in response to the immune system after transduction. This is the reason why there is a need for new techniques for T-cell modification that need either minimal manipulations ex vivo or no manipulation at all when administrated in vivo [90].

#### 3.3.2. Novel LV Pseudotypes Allow Efficient T Cell Transduction for Gene Therapy

Recently, several studies reported other LV pseudotypes (e.g., HF pseudotyped LVs) that have been used to improve the transduction efficiency in T cells without modifying their phenotype [42,46,58]. More recently, Bernadin et al. [43] have shown that BaEV pseudotyped LVs efficiently transduced naïve T cells and progenitor T cells, which upon engraftment in NOD/SCIDγC^−/−^ (NSG) mice developed into mature T-cell subpopulations that maintained these high transduction levels. They also observed that the T-cell lineage reconstitution was accelerated upon the injection of the progenitor T cells in comparison to HSCs. Therefore, these BaEV pseudotyped LVs have great potential for T cell-based SCID-X1 gene therapy.

Interestingly, a research team succeeded in engineering an LV that ensures selective and stable gene delivery to a specific T-cell subtype upon its in vivo administration. An LV was pseudotyped with a MV H glycoprotein incorporating a CD8-specific scFv, which allowed it to deliver the transgene specifically to CD8^+^ T cells by using the CD8 surface molecule as a receptor. They demonstrated that compared to VSV-G pseudotyped LVs, the CD8-specific LVs needed 5–10-fold less vector doses to obtain the same transduction efficiency [71]. The same team also designed an LV vector system which relied on pseudotyping with a MV H glycoprotein that was fused with a CD4-specific ankyrin repeat protein, creating a retargeted LV specific for entry into CD4 T cells. This CD4-LV efficiently targeted CD4^+^ T cells for gene delivery in human peripheral blood monocytes cells (PBMCs) (in vitro) and in humanized mouse models by systemic administration (in vivo). Moreover, they demonstrated that this LV can be used in the treatment of HIV infection by using it to deliver an inhibitor gene that prevents T cells of getting infected by HIV [48].

#### 3.3.3. Chimeric Antigen Receptor T Cells

To convert T cells into a powerful anti-cancer drug, chimeric antigen receptors (CAR), which recognize surface antigen on malignant cells, have been incorporated into T cells for immunotherapy [72,92]. They are considered an individualized cell therapy product as it requires harvesting of the patient’s T cells, which are then modified and expanded ex vivo to be re-infused. CAR-T cells are activated, expanded and kill target cells once they recognize the cancer antigen. The expression of the CAR has to be stable and these CAR T cells need to maintain their functionality and persist long term in vivo [72]. The main problem of in vivo gene delivery is the possibility of generating off-target cell LV transduction causing severe health risks and adverse effects [93]. Pfeiffer et al. [72] are the first to report the generation of human anti-CD19 CAR-T cells in vivo upon injection into humanized mice of an LV pseudotyped with a nipah G glycoprotein harboring an scFv directed against CD8 T cells. They showed that antiCD19-CAR^+^ CD8^+^ T cells generated in vivo possessed high cytotoxicity against CD19^+^ cells. Jamali et al. [94] modified the envelope protein of the nipah virus and fused it to a CD8-specific scFv, while the MV envelope protein was fused to a CD4-specific DARPin. These retargeted envelope gps were used to produce CD4^+^ or CD8^+^ T-cell specific LVs carrying CAR genes. This resulted in high selectivity for T lymphocytes, which facilitated and improved CAR T cell generation. Moreover, these authors showed that these CD8- and CD4-LVs allowed the generation of CAR-T cells in vivo due to their high specificity for T cells, excluding the need of T cell purification in the process of CAR-T manufacturing and possibly making this kind of therapy more accessible to a higher number of patients. However, improvements in CAR T-cell therapies are urgently needed since CAR T cell application is associated with toxicities, exhaustion, immune suppression, lack of long-term persistence and low CAR T-cell tumor infiltration. Major efforts to overcome these hurdles are currently on the way and are reviewed elsewhere [95].

Some of these improvements included transduction enhancers, such as cationic polymers, lipids or peptides. The most recent one is Vectofusin-1, a histidine-rich cationic amphipathic short peptide, which enhanced transduction with certain pseudotyped LVs, such as BaEV and GALV. Moreover, the addition of Vectofusin-1 did not impair the killing capability of these generated CAR-T cells [94].

### 3.4. B Cells as Gene Therapy Targets

B cells are not only interesting gene therapy targets for diseases associated with B cell dysfunction but also for immunotherapy. B-cell gene therapy is particularly appealing because B cells have the potential to induce specific immune activation. They are also important in the induction of tolerogenic antigen presenting cells, which has been demonstrated in animal models for autoimmune diseases such as diabetes [44]. In the case of B-cell gene therapy for monogenic diseases, hemophilia might be an important target disease to consider [87]. Hemophilia A and B are blood disorders caused by mutations in the clotting factor VIII and Factor IX (FVIII and FIX), which causes uncontrolled bleeding. Clinical trials for hemophilia have been using AAV to express either FVIII or FIX in the liver or muscle [96]. This approach, however, has numerous disadvantages revealed in the clinic, which emphasized the need for further improvement. Therefore, Lévy et al. [44] proposed not to transduce hepatocytes, which are the natural producers of FIX, but transduce B cells to allow them to express de novo factor FIX. BaEV pseudotyped LVs were compared to other envelope pseudotypes such as H/F, RD114 as well as the commonly used VSV-G. For the first time, BaEV pseudotyped LVs showed efficient transduction of both naïve and memory B cells [44] in similar way as the H/F previously described [42,57,58]. Lévy et al. showed that FIX encoding BaEV pseudotyped LVs transduced human B cells, which were able to home to the spleen and the bone marrow in immunodeficient NSG mice, where they differentiated into plasma cells that allowed expression of the human FIX in the blood stream at levels close to what is detected in healthy human subjects.

To reprogram B cells for ectopic antibody expression, it would be advantageous to include in the design of the vector the natural regulation of antibody expression, namely, the transition from the B-cell receptor (BCR) form to secreted immunoglobulins (Ig). With this objective in mind, Fusil et al. [97] designed an LV which coded for a human cross-neutralizing antibody against the envelope of the hepatitis C virus (HCV). It was the first study demonstrating that LVs allowed the physiological expression of a human antibody as these authors mimicked the natural mechanism of Ig maturation during the B-cell development in their vector design. To achieve efficient B-cell gene transfer, the LVs were pseudotyped with the BaEV envelope, previously described as one of the best candidate pseudotypes to transduce B cells [55], since it allowed efficient transduction of resting and stimulated human B cells [97].

### 3.5. Gene Therapy Using Natural Killer Cells

Natural killer (NK) cells kill virus-infected and tumor cells as part of the innate immune system without need of stimulation. They have also shown antitumor activity in HSC transplantation, and in the absence of T cells they facilitate HSC engraftment, combat infection and control the appearance of graft-versus-host disease (GvHD) [98,99,100]. Most of the research so far has been focused on redirecting T cells to combat cancer such as transforming them into CAR T cells (see Section 3.3.3). However, there is also an interest in developing similar approaches with NK cells. They are considered an appealing option because of their natural cytotoxic function and they avoid induction of GvHD. This means that NKs can be used in an allogenic setting. The main obstacle in using NKs has always been the low transduction efficiency. So far, the highest level of NK transduction achieved with VSV-G pseudotyped LVs was between 2% and 12% when cultured in presence of different cytokine cocktails [101]. Surprisingly, the use of BaEV pseudotyped LVs has overcome this obstacle and showed a 20-fold increase in the transduction efficiency when compared to the VSV-G pseudotyped LVs. What is more important, CAR expressing NK cells showed improvement in functionality and a higher ability to kill cancer cells [40,41]. This is proof of concept for the generation of CAR-NK cells in vitro, which may become powerful immunotherapeutic products in the future.

## 4. Gene Editing: A New Upcoming Tool for Gene Therapy

Gene editing is considered a type of genetic engineering, where the DNA is either inserted, deleted or replaced in the target cell genome using specific nucleases, by the creation of site-specific double-strand breaks (DSB). These DSB are corrected by the cellular DNA repair machinery, either by non-homologous end joining (NHEJ) or by direct homologous recombination (DHR), when adding DNA template [102]. The advantages of gene editing over gene addition are the ability to modify endogenous sequences specifically, maintaining the transcriptional regulation of the gene and reducing the risk of oncogene activation due to insertional mutagenesis [103,104].

There are various specific engineered nucleases used as gene editing tools such as ZFN, TALENs and more recently CRISPR/Cas9. CRISPR/Cas9 has been extensively used in the last decade due to its high specificity, activity and easy design [102,105]. Their applications cover various fields; biotechnology, biological investigation, human medicine applications and agricultural research [102].

In the context of gene therapy, HSCs are the targets of choice for gene editing-based therapies. The variability of efficiency of gene editing in cells is related to their repair pathway. It has been reported that adult primary cells use the error prone NHEJ instead of the DHR pathway due to their non-dividing stage. HIV infection is one of the most studied diseases using gene editing therapy approaches [106]. It has been the main objective to ablate the chemokine (C-C motive) receptor 5 (CCR5) in T cells, which has been demonstrated to protect against HIV infection. Deleting CCR5 in HSCs will give rise to lymphoid and myeloid linages resistant to HIV CCR5 strains providing long lasting immunity to the infection. This strategy has been tested using CRISPR/Cas9 multiplex guide RNAs (gRNA) and increased 10-fold the percentage of cleavage in CD34^+^ cells with no significant effect in their potency to differentiate into all hematopoietic lineages [107]. It has also been effectively used in targeting the interleukin-2 receptor gamma (IL2RG), which is the mutated gene in SCID-X1. Correction of the IL2RG in HSCs has shown their efficient engraftment and repopulation in NSG mice, which confirms gene edition in long-term repopulating HSCs [108].

Gene editing is being used to deplete the endogenous TCR, both the α and β chains, to avoid the GvHD and produce a universal CAR T cell. It has been shown that when this universal T cell is combined with depletion of other genes by CRISPR/Cas9, high anti-leukemic activity and no induction of GvHD were observed in the NSG mouse model [109].

For the previously mentioned studies, different methods for delivery the editing tools have been utilized such as electroporation, adenoviruses and LVs conferring different degrees of efficiency, toxicity and off-target effects. Ideally, the perfect gene editing tool should be able to deliver its cargo fast, precise, non-toxic and with low off-target effects. Recently, Mangeot et al. [110] described a vehicle for Cas9-sgRNA by which the ribonucleoproteins (RNPs) are packed into a virus like particle (VLP) from MLV, which they called “nanoblade”. They showed that these nanoblades were able to induce DSB more rapidly and efficiently than other delivery methods and they were able to deliver their cargo to not only immortalized cells but also to primary fibroblast, induced pluripotent stem cells and CD34^+^ cells both human and mouse. Interestingly, since these are VLPs, they can be pseudotyped as viral vectors with different envelope glycoproteins. Indeed, an efficient gene editing of primary hematopoietic cells was recently achieved, when the nanoblades were pseudotyped with VSV-G and BaEV glycoproteins, simultaneously [111]. Finally, the nanoblades can also be combined with DNA templates and mediate HR-based knock-in in cultured cells and primary cells.

## 5. Conclusions

Here, we summarized the development of several new LV pseudotypes that allow transduction in primary hematopoietic cells, which were difficult to modify genetically by the currently used LVs displaying VSV-G at their surface. BaEV-LVs and MV-LVs allow transduction of unstimulated T, B, NK and HSCs without affecting their phenotypes nor functional characteristics. LVs carrying Nippa virus gps retargeted to T cells might prove in the future to be efficient immune and gene therapy tools. Alternative LV pseudotypes will allow to address multiple fundamental questions and reveal the mechanism implicated in blood cell differentiation, function and transformation into malignant cells. At the moment, these lentiviral pseudotypes have not been utilized in clinical trials, but their production is being optimized for clinical applications.

## Figures and Tables

**Table 1:** Different viral envelope glycoproteins used for lentiviral vector pseudotyping and their cell tropism.

Pseudotypes	Original Virus	Receptor	Cell Tropism	Efficiency	References
VSV-G	Vesicular stomatitis virus	LDL-R	Broad in non-primary cells	High	[29]
BaEV	Baboon endogenous retrovirus	ASCT-1 ASCT-2	CD34^+^ cells	30%	[36]
			Naïve T cells	Up to 80%	[39,40,41]
			Naïve B cells	40%	[42]
			Memory B cells	20%	[42]
			Natural killer	40%	[39,40,41]
			Early thymocytes	Up to 80%	[39,40,41]
RD114	Feline endogenous retrovirus	ASCT-2	Naïve T cells	Up to 60%	[43]
			Naïve B cells	Up to 30%	[44]
H/F	Measles virus	SLAM CD46	CD34^+^ cells		[30,45]
			Resting memory T cells		[46]
			Naïve T cells	Up to 50%	[46]
			Quiescent B cells		[42,46]
			Resting HSCs	Up to 70%	[47]
			Dendritic cells		[47,48]
G/F	Nipah virus	EphinB2/B3	Pericytes	20–40%	[49,50]
			Tumor endothelium		[49,50]
COCV	Cocal virus	LDL-R	Stimulated CD34^+^ cells	Up to 80%	[32]
